# Succinic Acid‐Induced Macrophage Endocytosis Promotes Extracellular Vesicle‐Based Integrin Beta1 Transfer Accelerating Fibroblast Activation and Sepsis‐Associated Pulmonary Fibrosis

**DOI:** 10.1002/advs.202507411

**Published:** 2025-09-02

**Authors:** Wenyu Yang, Ri Tang, Yang Zhou, Jinquan Zhang, Shuya Mei, Yawen Peng, Xi Huang, Shunpeng Xing, Yuan Gao, Qiaoyi Xu, Zhengyu He

**Affiliations:** ^1^ Department of Critical Care Medicine Renji Hospital Shanghai Jiao Tong University School of Medicine Shanghai 200127 China; ^2^ Key Laboratory of Anesthesiology (Shanghai Jiao Tong University) Ministry of Education Shanghai 200127 China; ^3^ Department of Anesthesiology National Cancer Center/National Clinical Research Center for Cancer/Cancer Hospital and Shenzhen Hospital Chinese Academy of Medical Sciences and Peking Union Medical College Shenzhen 518116 China

**Keywords:** extracellular vesicles, integrin β1, macrophage‐fibroblast communication, pulmonary fibrosis, sepsis

## Abstract

Sepsis‐associated pulmonary fibrosis (SAPF) is a life‐threatening condition driven by persistent fibroblast activation and excessive extracellular matrix (ECM) deposition. While metabolic reprogramming, profibrotic extracellular vesicles (EVs), and integrin activation are implicated in pulmonary fibrosis, their interplay remains unclear. This study reveals that succinic acid, a product of glycometabolic reprogramming, promotes macrophage‐mediated endocytosis, driving the release of profibrotic EVs. These EVs transfer integrin beta1 (ITGβ1) from macrophages to fibroblasts, activating fibroblasts and advancing SAPF. Through Single‐cell RNA sequencing (scRNA‐seq), proteomics, immunofluorescence, and electron microscopy, the critical role of EV‐mediated ITGβ1 transfer in macrophage‐fibroblast communication is identified. Knockdown of ITGβ1 or Alix, a mediator of multivesicular bodies (MVBs) biogenesis, inhibited profibrotic EVs formation and alleviated SAPF. These findings highlight a novel mechanism in that the transfer ITGβ1 via EVs plays a critical role in macrophage‐fibroblast communication, representing a novel mechanism underlying SAPF. Targeting EV‐mediated ITGβ1 transfer can provide a promising therapeutic strategy to alleviate the progression of SAPF.

## Graphical abstract

Lipopolysaccharide (LPS) stimulates the production of succinic acid in lung tissue, which promotes macrophages endocytosis and the formation of multivesicular bodies (MVBs). These MVBs release profibrotic extracellular vesicles (EVs), facilitating the transfer of integrin beta1 (ITGβ1) transfer and subsequently activating fibroblasts, thereby contributing to the development of sepsis‐associated pulmonary fibrosis (SAPF).

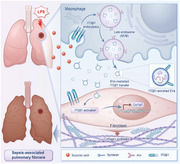

## 1 Introduction

Sepsis is a clinical syndrome with high mortality caused by infection and manifested as a dysregulated host response that leads to life‐threatening organ dysfunction.^[^ ^1^ ^]^ When sepsis affects the lungs, the primary target organ, the structure and function are impaired to varying degrees. The pathological features include protein‐rich pulmonary edema and hyaline membrane formation in alveolar exudate, resulting from increased pulmonary microvascular permeability. This triggers a pulmonary inflammatory response, which can lead to acute respiratory distress syndrome (ARDS).^[^ ^2^ ^]^ In severe cases, it may progress to pulmonary fibrosis and eventually respiratory failure, which is associated with a poor prognosis and high mortality.^[^ ^3^, ^4^ ^]^ Unfortunately, there are no effective clinical treatments for sepsis‐associated pulmonary fibrosis, making it urgent to investigate the underlying mechanisms and develop target therapies.^[^ ^5^, ^6^, ^7^ ^]^

Intercellular communication drives microenvironmental changes that influence the proliferation and activation of fibroblasts, a progress essential for the development of pulmonary fibrosis.^[^ ^8^, ^9^ ^]^ Such signaling occurs through the release of soluble molecules or the transfer of extracellular vesicles (EVs). EVs encompass various subtypes, including exosomes, microvesicles (MVs), ectosomes, oncosomes, and apoptotic bodies.^[^ ^10^, ^11^ ^]^ These vesicles serve as carriers of bioactive molecules, such as proteins, nucleic acids, lipids, and metabolites, which are necessary for the occurrence of biological activity.^[^ ^12^, ^13^ ^]^ Integrins, a family of transmembrane heterodimeric receptors, mediate cell adhesion and signal transduction.^[^ ^14^, ^15^ ^]^ Located on the surface of EV membranes, integrins endow EVs with the capacity to target specific cells or organs.^[^ ^16^ ^]^ Previously, we reported that integrin subunit β3 plays a pivotal role in the progression of mechanical ventilation (MV) and lipopolysaccharide (LPS)‐induced pulmonary fibrosis.^[^ ^17^, ^18^ ^]^ Thus, further investigation into the transport pathways of integrins on the EVs membranes is vital to elucidating the pathogenesis of SAPF.

Glycometabolic reprogramming occurs in various cell types, including fibroblasts, epithelial cells, and macrophages, and promotes pulmonary fibrosis by modulating autophagy, cell proliferation, apoptosis, extracellular matrix (ECM) synthesis, and other processes.^[^ ^19^ ^]^ Interestingly, an increasing number of studies have confirmed that metabolites involved in glycolysis, tricarboxylic acid cycle (TCA cycle), and fatty acid metabolism undergo dynamic alterations during pulmonary fibrosis.^[^ ^20^ ^]^ Succinic acid, a key metabolite of the TCA cycle, plays a critical role in the progression of glucose metabolic reprogramming in macrophages^[^ ^21^ ^]^ and promotes transforming growth factor (TGF)‐induced activation of normal human lung fibroblasts.^[^ ^22^ ^]^ We have verified that integrin β3 activation regulate glycometabolic reprogramming and fibroblasts activation during pulmonary fibrosis, but how succinic acid dynamics contribute to the transfer of integrin beta1 (ITGβ1) and pathogenesis of SAPF remains unclear.

We hypothesized that succinic acid‐induces macrophages to release profibrotic EVs, mediating ITGβ1 transfer and subsequently activating fibroblasts during SAPF. In this study, we used multi‐omics approaches and diverse experimental techniques in vivo and in vitro to investigate the role of succinic acid and EV‐mediated integrin transfer in macrophage‐fibroblast interactions and fibroblasts activation. Our findings elucidated the interconnections among these factors, providing new insights into the molecular mechanisms underlying SAPF.

## 2 Results

### 2.1 SAPF is Accompanied by Elevated Succinic Acid Levels and Profibrotic EVs Secretion

**Figure 1:**
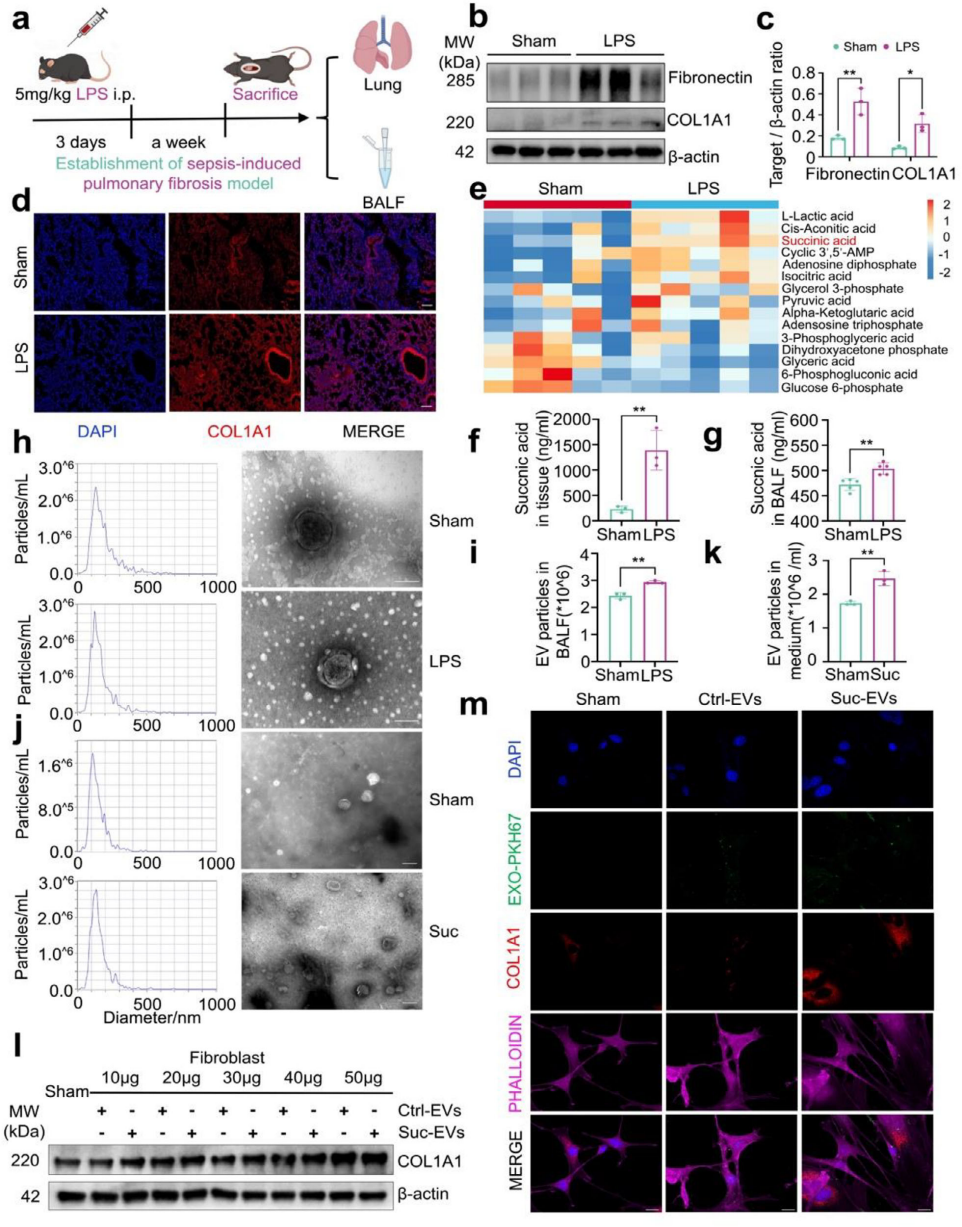
Elevated succinic acid and profibrotic EVs secretion in LPS‐induced pulmonary fibrosis. a) Schematic showing LPS‐induced pulmonary fibrosis progression. LPS was administered intraperitoneally for 3 consecutive days and samples were collected one week later. b–c) Western blot and the corresponding quantitative analysis to detect fibrosis markers Fibronectin and COL1A1 after LPS treatment (Mean ± s.d., ^*^ *P* < 0.05, ^**^ *P* < 0.01, unpaired *t*‐test, *n* = 3). d) Immunofluorescence analysis of lung tissues from the sham and LPS groups in mice revealed the expression and distribution of COL1A1.Scale bar=200 µm. e) Metabolomics to analyze the changes in various metabolites in the lung tissues of mice, with the results presented in the form of a heatmap (*n* = 5). f–g) Succinic acid metabolic profiles measurement in lung tissues (*n* = 3) and BALF (*n* = 5) from sham and LPS groups (Mean ± s.d., ^**^ *P* < 0.01, unpaired *t*‐test). h–i) TEM images, NTA analysis and the corresponding statistics of EVs in BALF. Scale bar=100 nm. j–k) Purified EVs derived from macrophages were determined using TEM images, NTA analysis (Mean ± s.d., ^**^ *P* < 0.01, unpaired *t*‐test). Scale bar=100nm. l) Western blot to detect the expression of COL1A1 in fibroblasts stimulated with 10–50 µg of macrophages‐derived EVs. “Ctrl‐EVs” = EVs released by the control group macrophages, “Suc‐EVs” = EVs released by the macrophages stimulated with succinic acid. m) Immunofluorescence analysis of COL1A1 in fibroblasts stimulated with 40 µg EVs. PKH67‐labeled EVs appeared as green punctate structures and phalloidin (magenta)‐labeled F‐actin represents the cell outline. Scale bar=10 µm. Suc=Succinic acid. The partial results presented in Figure 2 and 5 are derived from the samples generated during present experiment, which were displayed in relevant Figures to adhere to the logical coherence required for the presentation of the findings.

To establish the model of SAPF, LPS was administered intraperitoneally at 5 mg/kg /day for three consecutive days, as previously reported (**Figure** **1**a). One week after drug administration, the experimental mice were sacrificed, and lung tissues were harvested for further studies.  Hematoxylin and eosin (H&E) staining showed significant alveolar congestion and interstitial inflammatory cell infiltration in the LPS group compared to the sham group. Meanwhile, Masson staining revealed a marked increase in peribronchial collagen fibers in the LPS group (Figure S1a, Supporting Information). Consistent with these pathological changes, the expression of collagen type 1 alpha 1 chain (COL1A1) and fibronectin, key markers associated with fibroblast activation during pulmonary fibrosis, was significantly elevated in the LPS group (Figure 1b,c). Moreover, COL1A1 was primarily localized in the lung interstitum, with additional localization observed in the surrounding regions of the lung tracheae (Figure 1d).

To measure succinic acid levels in lung tissues, samples were prepared as homogenates and analyzed using HPIC‐MS/MS. As shown in Figure 1e, LPS induced a substantial increase in succinic acid, L‐Lactic acid, and adenosine diphosphate levels. Similarly, LC‐MS confirmed that LPS promoted the generation of succinic acid not only in lung tissues but also in BALF (Figure 1f,g; Figure S1b,c, Supporting Information). During the development of pulmonary fibrosis, various cell types, including macrophages, can influence succinic acid levels through metabolic reprogramming. Enrichment analysis showed that macrophages are involved in the regulation of several metabolic pathways, including oxidative phosphorylation.^[^ ^23^, ^24^ ^]^ Additionally, pathways related to the synthesis and release of EVs can also be enriched (Figure S2a,b, Supporting Information). Flow cytometry experiments revealed that macrophages play a significant role in SAPF (Figure S1d, Supporting Information).

To investigate EVs production during SAPF, TEM confirmed the presence of vesicular lipid bilayer structures in BALF EVs, while nanoparticle tracking analysis (NTA) showed a significant increase in EVs numbers in the LPS group (Figure 1h,i; Figure S2c, Supporting Information). Considering the role of succinic acid in promoting EVs release, subsequent in vitro experiments were conducted. Macrophages were stimulated with 5 mmol/L succinic acid to enhance EVs secretion (Figure 1j,k; Figure S2d, Supporting Information). EVs were purified from macrophages by ultracentrifugation and used to treat fibroblasts. Fibroblast activation showed a dose‐dependent relationship with EVs concentrations (Figure 1l). When fibroblast cell lines and primary cells were stimulated with 40 µg of Suc‐EVs, significant activation of COL1A1 was observed via immunofluorescence analysis (Figure 1m; Figure S2e, Supporting Information). Taken together, these findings suggest that LPS promotes succinic acid release, which induces macrophage‐derived profibrotic EVs, further leading to fibroblast activation during SAPF.

### 2.2 Macrophages Transfer ITGβ1 to Activate Fibroblasts Through the Release of Profibrotic EVs

**Figure 2:**
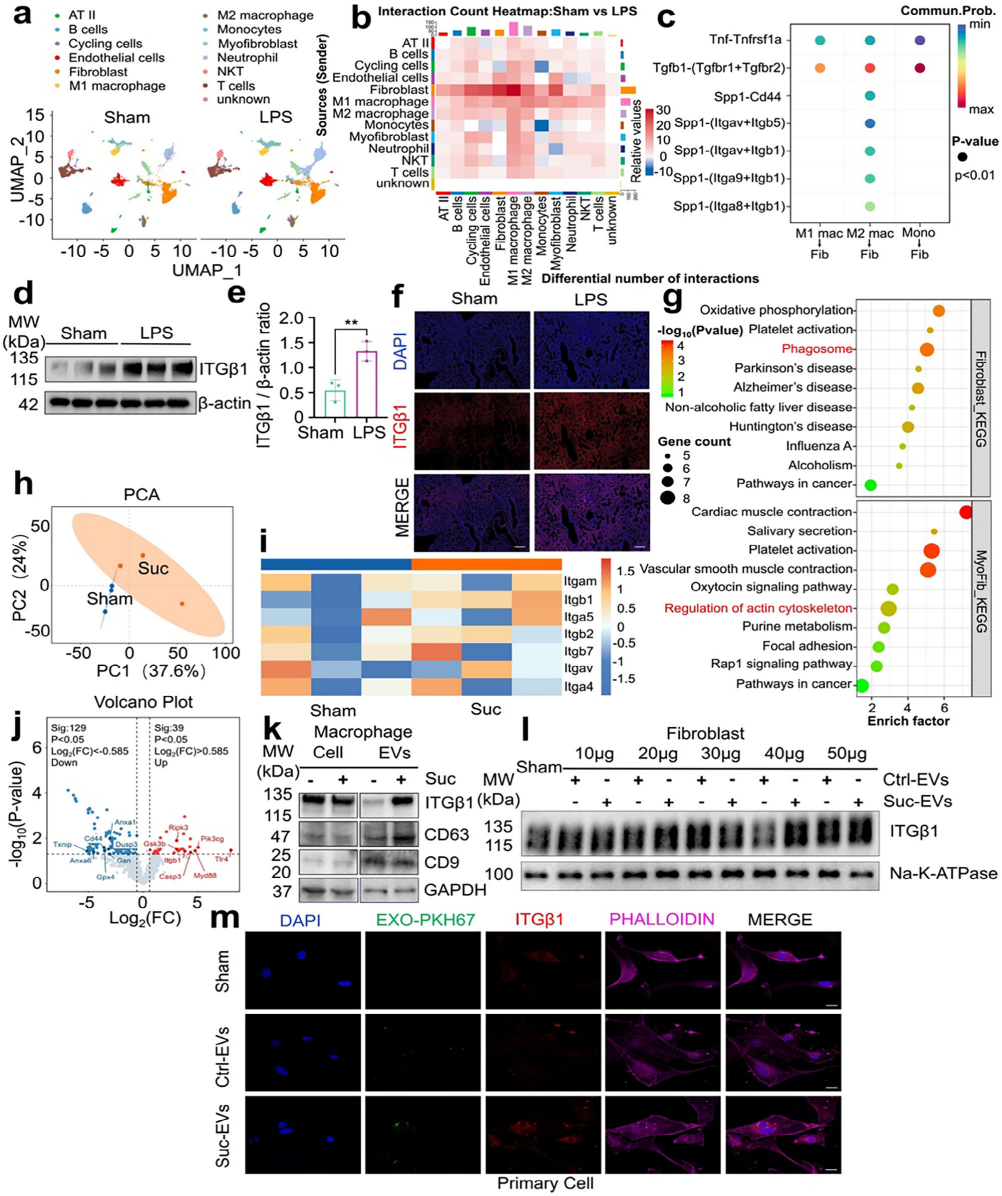
Macrophage‐derived profibrotic EVs transfer ITGβ1 to activate fibroblasts. a) RNA expression data from lung tissues of sham and LPS groups were analyzed using Uniform Manifold Approximation and Projection (UMAP) and clustering techniques. b) Differential interaction count heatmap between cells, as recommended by CellChat. c) Bubble plots further quantitatively visualized major intercellular signaling pathway interactions, including those from macrophages to fibroblasts and monocytes to fibroblasts. d,e) Western blot and the corresponding quantitative analysis to determine the expression of ITGβ1 from sham and LPS‐stimulated lung tissues (Mean ± s.d., ^**^ *P*< 0.01, unpaired *t*‐test, *n* = 3). f) Immunofluorescence analysis of ITGβ1 w/wo LPS stimulation. Scale bar=200 µm. g) KEGG analysis of pathways enriched by upregulated differentially expressed genes (DEGs) in fibroblasts and myofibroblasts. h) Principal component analysis (PCA) plot demonstrated the spatial relationship of cell proteomic profiles of EVs from macrophages. i) The heatmap showed the relative protein expression levels of 7 integrin family members in EVs from the sham and Suc groups. In each enrolled case, red indicates upregulated proteins, whereas blue indicates downregulated proteins (n = 3). j) Volcano plot of differential proteins between the sham and the Suc groups. The x‐axis represents the fold change of differential proteins (log2 value), whereas the y‐axis represents the p‐value (‐log10 value). Grey indicates non‐significant proteins, red denotes upregulated proteins, while blue denotes downregulated proteins. k) Western blot of a cell lysate and an EVs preparation. l) Western blot analysis to examine the expression levels of ITGβ1 in fibroblasts stimulated with 10–50 µg of EVs derived from macrophages. m) Immunofluorescence analysis of ITGβ1 in primary fibroblasts stimulated with 40 µg EVs. Scale bar = 10 µm.

The above observations suggest that elevated succinic acid levels in lung tissues exacerbate pulmonary fibrosis by promoting macrophage‐derived profibrotic EVs that activate fibroblasts. To explore the cellular dynamics during the pathogenesis of SAPF, single‐cell RNA sequencing (scRNA‐seq) was conducted on lung tissues from sham and LPS groups. Data dimensionality was reduced using uniform manifold approximation and projection (UMAP), and cell clustering analysis was performed based on marker gene identification (**Figure** **2**a). Using CellChat, we analyzed cell–cell communication between macrophages and fibroblasts. This tool quantified interaction probabilities and incorporated key ligand‐receptor interactions.^[^ ^25^ ^]^ LPS exposure was found to enhance the interactions between macrophages and fibroblasts. Additionally, LPS strengthened macrophage interactions with myofibroblasts, a cell population originating from fibroblast‐to‐myofibroblast transition (FMT). These cells contribute to pulmonary fibrosis progression by releasing ECM components (Figure 2b; Figure S4b, Supporting Information). Bubble plots quantitatively illustrated that there is a significant interaction between M2 macrophages and fibroblasts, where SPP1 acts as a ligand that can bind to integrins, particularly ITGβ1. This binding plays an important role in cell adhesion and signal transduction.^[^ ^26^ ^]^ (Figure 2c). Furthermore, increased ITGβ1 expression in lung tissues in response to LPS was confirmed (Figure 2d–f; Figure S3a, Supporting Information). KEGG pathway enrichment analysis revealed that fibroblasts form phagosomes and regulate the actin cytoskeleton to enhance ECM secretion (Figure 2g).

In vitro experiments elucidated the mechanisms underlying this process. Proteomics of macrophage‐derived EVs revealed distinct clustering between groups in principal component analysis (PCA) plots (Figure 2h) and differential protein abundance related to the integrin family visualized by heatmaps (Figure 2i). Succinic acid exposure notably altered EVs composition, with ITGβ1 significantly enriched compared to other integrin family members. Volcano plots further highlighted six profibrotic genes are upregulated, eight antifibrotic genes are downregulated, and itgb1 is upregulated. (Figure 2j). Western blot analysis corroborated increased ITGβ1 levels in EVs but not in macrophages themselves (Figure 2k). Fibroblasts stimulation with varying EVs doses demonstrated elevated ITGβ1 expression in a dose‐dependent manner, with 40 µg producing the most pronounced effects (Figure 2l). Immunofluorescence analysis yielded consistent results, confirming ITGβ1 upregulation in fibroblasts (Figure 2m). Based on the above experimental results, we speculated that the upregulation of ITGβ1 in fibroblasts is partly transferred from profibrotic EVs secreted by macrophages. To clarify this hypothesis, we tagged ITGβ1 with a HaloTag and employed membrane‐permeant dyes and a self‐fluorescent mCherry tag to image the route of ITGβ1.^[^ ^27^ ^]^ As shown in Figure S3b,c (Supporting Information), we observed that after stimulating fibroblasts with EVs treated with succinic acid, which labeled with PKH67 or PKH26 dyes, co‐localized with ITGβ1 tagged with mCherry or Halo in fibroblasts, demonstrating that this portion of ITGβ1 was transferred and activated by EVs. Together, these results demonstrate that macrophages mediate fibroblast activation by transferring ITGβ1 through profibrotic EVs.

### 2.3 Succinic Acid‐Stimulated EVs Activate Fibroblasts Through the Transfer of ITGβ1, Thereby Contributing to the Progression of SAPF

**Figure 3:**
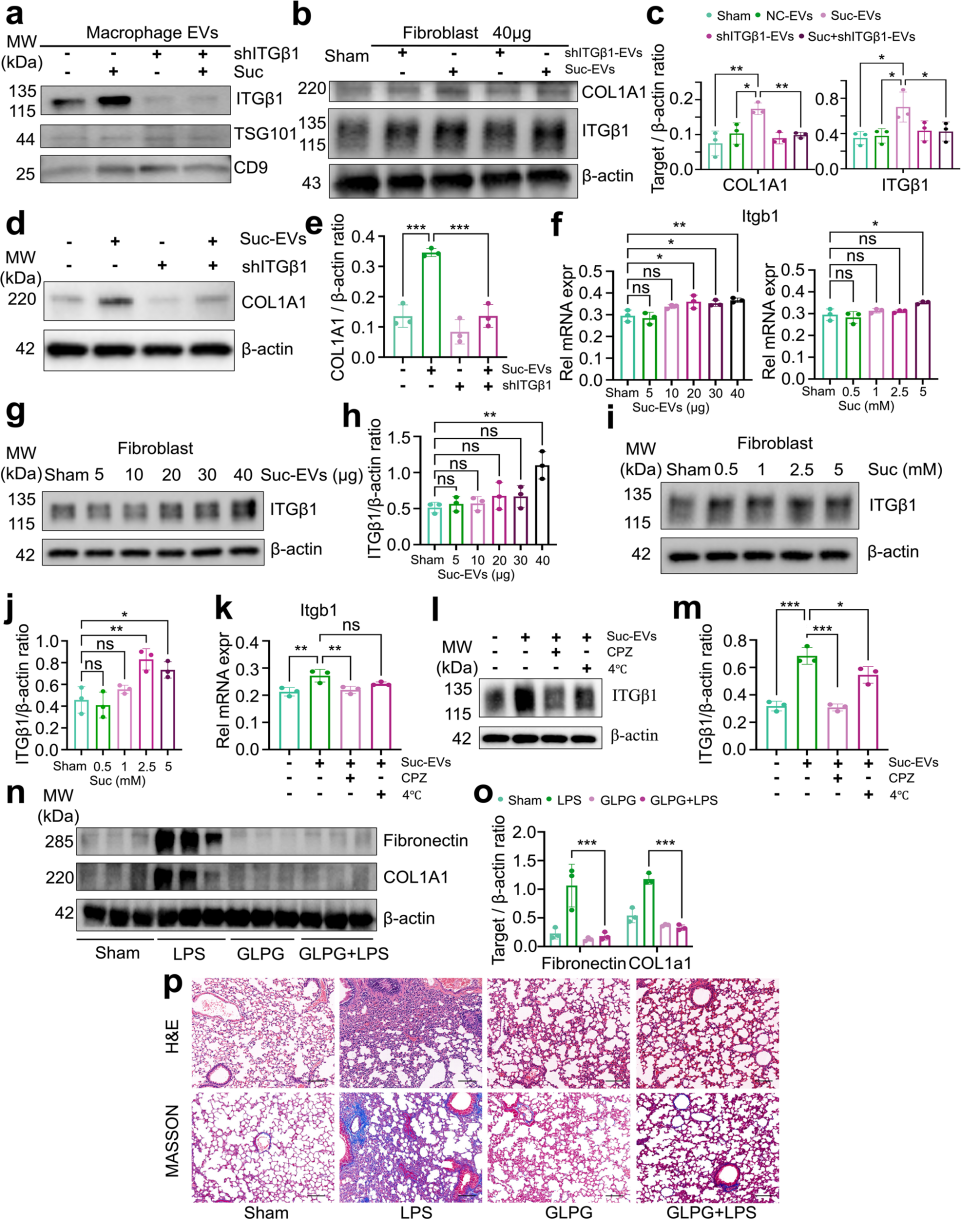
ITGβ1 is critical for profibrotic EVs secretion, LPS‐induced fibroblast activation, and pulmonary fibrosis. Western blot analysis to detect the expression levels of ITGβ1 in a) EVs derived from macrophages w/wo Itgb1 knockdown combined with succinic acid treatment and b,c) fibroblasts treated with 40 µg of the corresponding EVs and quantitative analysis. “shITGβ1‐EVs” = EVs released by macrophage stable cell lines with ITGβ1 knockdown. “Suc+shITGβ1‐EVs” = EVs released by the ITGβ1 knockdown stable cell lines stimulated with succinic acid. (Mean ± s.d., ^*^ *P*< 0.05, ^**^ *P*< 0.01, One‐Way ANOVA, n = 3). d,e) Western blot analysis to detect the expression of COL1A1 in fibroblasts treated with 40 µg of the Suc‐EVs w/wo Itgb1 knockdown and quantitative analysis. (Mean ± s.d., ^***^ *P*< 0.001, One‐Way ANOVA, *n* = 3). f) RT‐PCR analysis to detect mRNA levels of Itgb1 in fibroblasts stimulated with different concentrations of either Suc‐EVs or succinic acid. (Mean ± s.d., ^*^ *P*< 0.05, ^**^ *P*< 0.01, “ns” indicating not significant. One‐Way ANOVA, n = 3). g–j) Western blot and quantitative analysis to detect the aforementioned indicator. k–m) RT‐PCR and western blot to quantitative analysis the Itgb1 in fibroblasts following Suc‐EVs treatment w/wo 10 µm chlorpromazine (CPZ) or 4 °C pretreated for 1 h. (Mean ± s.d., ^*^ *P*< 0.05, ^***^ *P*< 0.001. n–o) Western blot and the corresponding quantitative analysis to assess Fibronectin and COL1A1 levels following LPS treatment w/wo 100 mg/kg GLPG0187. (Mean ± s.d., ^***^ *P*< 0.001, Two‐Way ANOVA, n = 3). p) H&E and Masson staining of LPS‐treated lung tissues, w/wo GLPG0187 for 3 days. The images are representative of more than three mice per group. Scale bar=100 µm.

To further investigate the essential role of ITGβ1 during the process of macrophages releasing profibrotic EVs, we knocked down the mRNA expression and protein levels of ITGβ1 in macrophages (Figure S3d,e, Supporting Information). Notably, ITGβ1 levels in EVs produced by succinic acid‐stimulated macrophages were significantly reduced following ITGβ1 knockdown (**Figure** **3**a). Additionally, the activation of fibroblasts and the elevated ITGβ1 expression induced by succinic acid were partially attenuated (Figure 3b,c). To further confirm the pivotal role of EVs‐derived ITGβ1 in the activation of fibroblasts, we performed a knockdown of ITGβ1 in fibroblasts (Figure S3f, Supporting Information) and subsequently stimulated them with 40 µg of EVs released by macrophages in response to succinic acid (Suc‐EVs) for 24 h. The findings indicated that stimulation with Suc‐EVs resulted in an increase expression of COL1A1, which was significantly diminished following the knockdown ITGβ1. Notably, upon subsequently stimulation with Suc‐EVs, the expression of COL1A1 was restored to levels comparable to those observed in the control group (Figure 3d,e). To eliminate the possibility that succinic acid itself may also induce an increase in ITGβ1 expression in fibroblasts, we compared the changes in mRNA and protein levels after stimulating fibroblasts with Suc‐EVs versus directly using succinic acid. As shown in Figure 3f–j, in comparison to stimulating fibroblasts directly with succinic acid, the stimulation with Suc‐EVs resulted in a significant increase in ITGβ1 expression, with changes in protein levels being more pronounced than those at the mRNA level. This phenomenon suggests that the increase in ITGβ1 expression in fibroblasts primarily originates from the transfer of exogenous EVs secreted by macrophages stimulated by succinic acid. Additionally, we found that inhibiting the uptake of EVs in fibroblasts resulted in varying degrees of reduction in ITGβ1 expression (Figure 3k–m). Based on these findings, we hypothesized that targeting ITGβ1 might serve as a potential therapeutic strategy for SAPF. To test this, we evaluated the effect of GLPG0187, a broad‐spectrum integrin receptor antagonist, in the SAPF mouse model. Consistent with our hypothesis, GLPG0187 treatment suppressed fibroblast activation, inflammatory cell infiltration, and collagen deposition in lung tissues (Figure 3n–p). Taken together, these results confirm that succinic acid‐stimulated profibrotic EVs generated by macrophages activate fibroblasts through the transfer of ITGβ1, which plays a crucial role in the development of SAPF.

### 2.4 Succinic Acid Promotes MVB Formation and EVs Secretion Through Endocytosis in Macrophages

**Figure 4:**
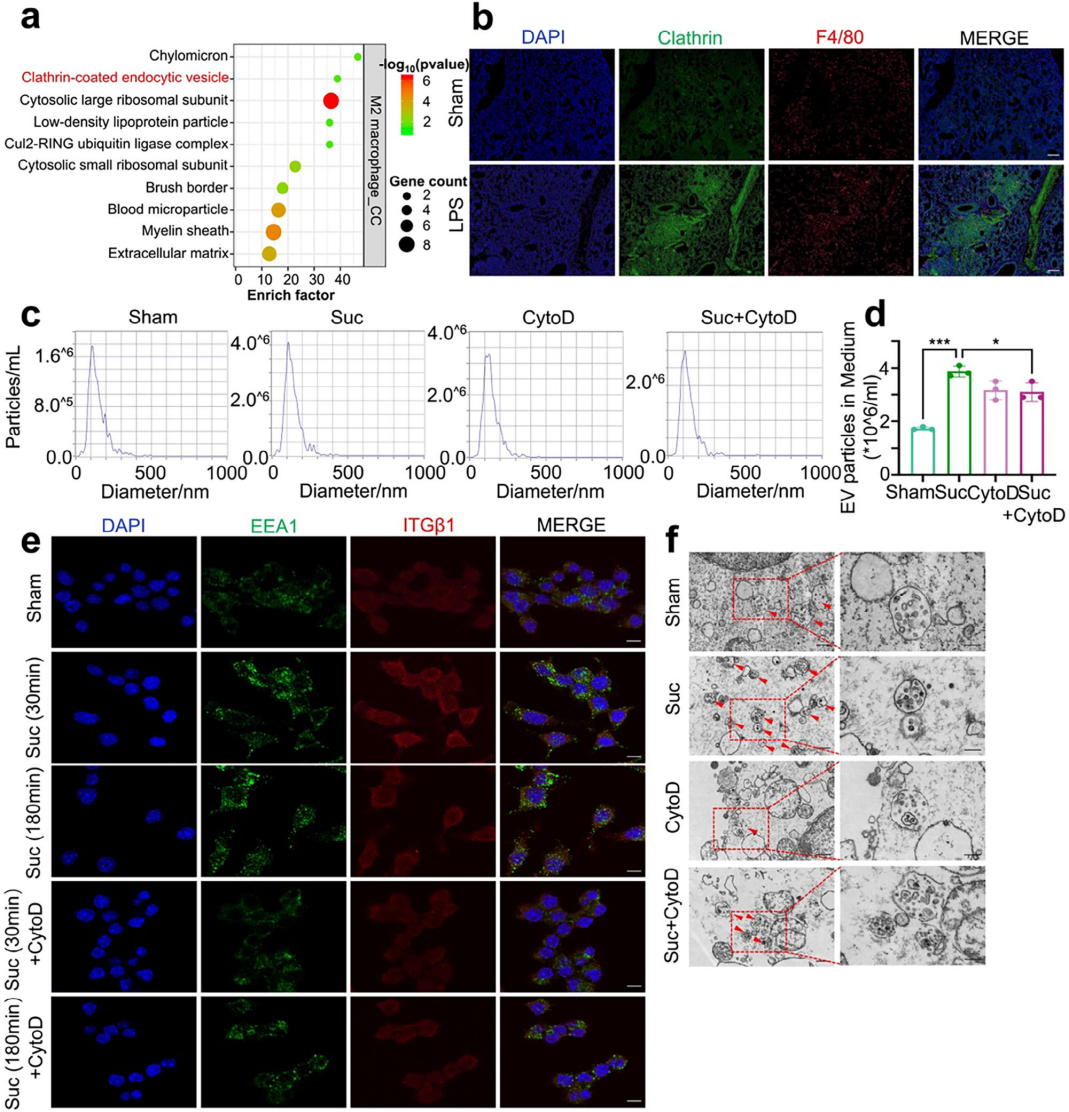
Succinic acid promotes ITGβ1 endocytosis, inducing MVBs formation and EVs secretion in macrophages. a) GO analysis of upregulated DEGs derived from M2 macrophages in the lung tissues of mice stimulated with LPS. b) Immunofluorescence analysis to detect the relative expression and distribution of clathrin and macrophage marker F4/80. Scale bar=200 µm. c,d) NTA and the corresponding quantitative analysis to assess the size distribution and quantity of EVs in macrophages following succinic acid treatment, w/wo 10 µm cytoD pretreatment for 1 h. (Mean ± s.d., ^*^ *P*< 0.05, ^***^ *P*< 0.001, One‐Way ANOVA, n = 3). e) Immunofluorescence analysis of MVBs marker EEA1 and ITGβ1 in macrophages stimulated with 5 mm succinic acid for 30 or 180 min, w/wo cytoD pretreatment. Scale bar=10 µm. f) TEM images were performed to visualize the morphology and quantity of MVBs and ILVs in macrophages after succinic acid treatment, w/wo cytoD pretreatment. Red arrows mark MVBs. Scale bar=0.5/0.25 µm (magnified view).

While previous results established the importance of ITGβ1, the detailed mechanisms underlying its role in EVs secretion required further exploration. GO analysis revealed the enrichment of clathrin‐coated endocytic vesicles, suggesting that LPS might impact internalization and migration progress (**Figure** **4**a). The results of the GO enrichment for macrophage‐derived EVs further support this conclusion (Figure S4a, Supporting Information). Clathrin‐mediated endocytosis (CME), the most common form of endocytosis, facilitates the packaging of EVs cargo proteins into the cytoplasmic structures. Immunofluorescence analysis of mouse lung tissues indicates that clathrin may play a role in LPS‐induced process (Figure 4b).

By using Cytochalasin D (CytoD), an actin‐dependent phagocytosis inhibitor, we found that inhibiting endocytosis significantly decreased EVs secretion in macrophages (Figure 4c,d). MVBsare organelles defined by a single membrane, which contain intraluminal vesicles (ILVs), the precursor structure of EVs.^[^ ^28^ ^]^ Interestingly, short‐term succinic acid treatment (30 and 180 min) upregulated the expression of early endosomal markers Rab5 and EEA1. However, these effects were attenuated by cytoD treatment (Figure 4e; Figure S4e, Supporting Information). Electron microscopy further demonstrated increased numbers of MVBs and ILVs per cell under succinic acid stimulation, which were reduced after CytoD application (Figure 4f). Collectively, these findings indicate that succinic acid can induce the formation of MVBs in macrophages and promote the secretion of profibrotic EVs. The use of the endocytosis inhibitor CytoD can inhibit these processes.

### 2.5 Syntenin‐1 and Alix Mediated Endocytosis‐Driven MVBs Formation and EVs Secretion

**Figure 5:**
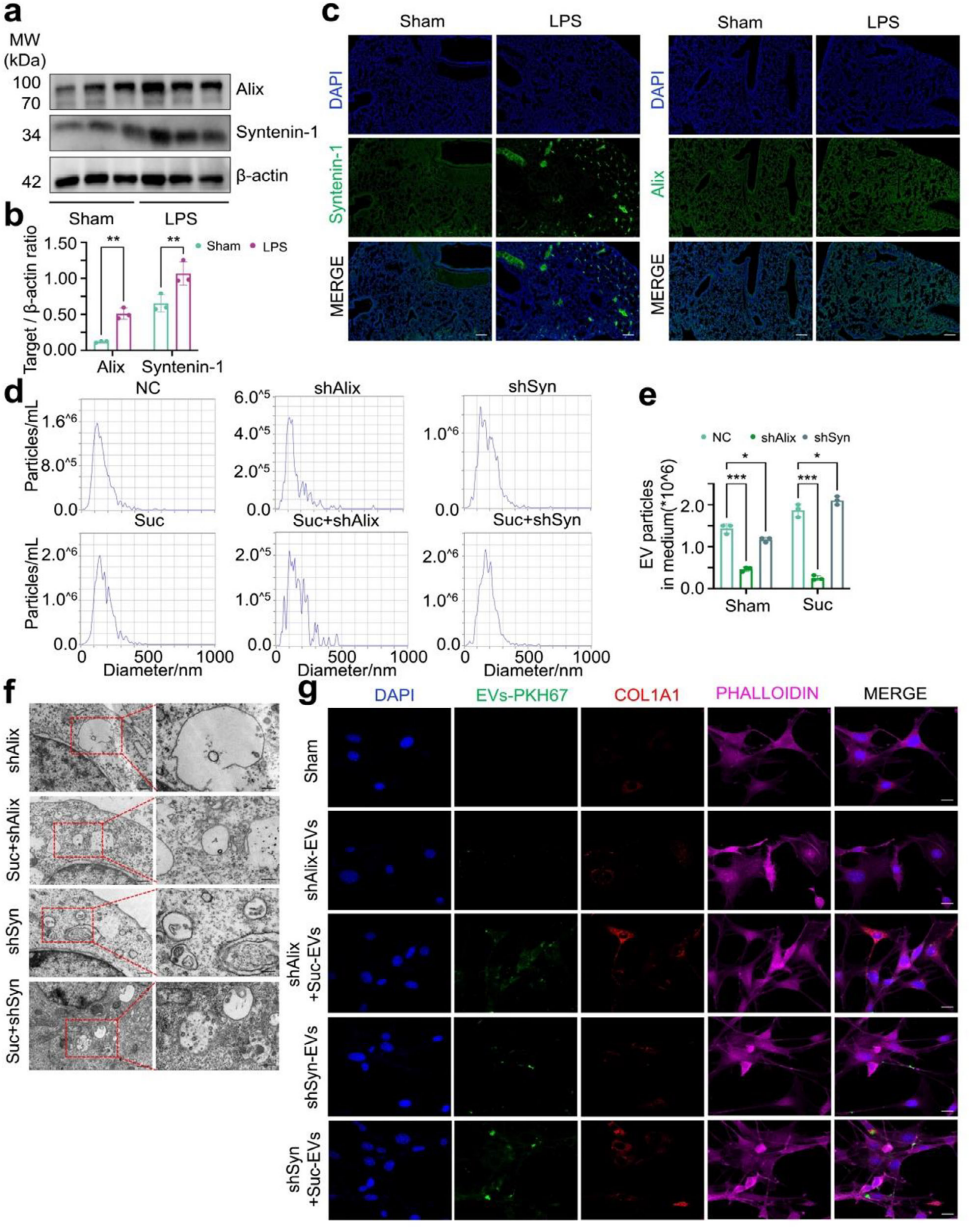
Syntenin‐1 and Alix regulate endocytosis‐driven MVBs formation and EVs secretion. a,b) Western blot and the corresponding quantitative analysis to determine the expression levels of Alix and Syntenin‐1 from sham and LPS‐stimulated lung tissues (Mean ± s.d., ^**^ *P*< 0.01, Two‐Way ANOVA, n = 3). c) Immunofluorescence was used to analyze Alix and Syntenin‐1 expression levels and distribution in mice lung tissues w/wo LPS stimulation. The images are representative of more than three mice per group. Scale bar=200 µm. d,e) NTA and the corresponding quantitative analysis to assess the size distribution and quantity of EVs in macrophages following 5 mM succinic acid treatment, w/wo Alix or Syntenin‐1 knockdown (Mean ± s.d., ^*^ *P*< 0.05, ^***^ *P*< 0.001, Two‐Way ANOVA, n = 3). NC = Negative Control. f) TEM images to analyze the morphology and quantity of MVBs and ILVs in macrophages after succinic acid treatment, w/wo Alix or Syntenin‐1 knockdown. Scale bar=0.5/0.25 µm (magnified view). g) Immunofluorescence was conducted to detect COL1A1 in fibroblasts stimulated with 40 µg EVs, w/wo Alix or Syntenin‐1 knockdown. Scale bar=10 µm.

The endosomal‐sorting complex required for transport (ESCRT) machinery plays a pivotal role in membrane budding and the sorting of ubiquitylated membrane proteins into specific domains of endosomes. This process facilitates endosomal invagination through ESCRT‐0, ‐I and ‐II complexes.^[^ ^29^ ^]^ Syntenin‐1, a cytosolic adaptor, links syndecans to activated leukocyte cell adhesion molecule (Alix), a key auxiliary component of the ESCRT machinery, regulates endosomal membrane budding and abscission, consequently driving the synthesis and release of EVs.^[^ ^30^, ^31^ ^]^ To investigate the roles of Syntenin‐1 and Alix in the production of profibrotic EVs, we analyzed their expression levels in lung tissues from both the sham and LPS groups. As shown in (**Figure** **5**a–c), both Alix and Syntenin‐1 showed increased expression and fluorescence intensity in the LPS group compared to the sham group, and they exhibited colocalization with pulmonary macrophages (Figure S5a, Supporting Information). However, in macrophages, succinic acid increased the mRNA levels of Alix and Syntenin‐1 without directly increasing their protein expression levels (Figure S5b,c, Supporting Information). To further validate their importance, we knocked down the mRNA expression and protein levels of Alix and Syntenin‐1 in macrophages (Figure S5d,e, Supporting Information). The use of the EVs release inhibitor GW4869 significantly decreased the secretion of EVs from macrophages (Figure S5f,g, Supporting Information). However, the knockdown of Alix led to a more pronounced reduction in EVs secretion compared to the knockdown of Syntenin‐1 (Figure 5d,e, Supporting Information). Electron microscopy analysis revealed that MVB structures were more disrupted following Alix knockdown compared to Syntenin‐1 knockdown (Figure 5f). Finally, EVs derived from macrophages were used to stimulate fibroblasts, and fibroblast activation was found to be concurrently reduced (Figure 5g). Collectively, these findings indicate that both Syntenin‐1 and Alix mediated MVBs formation and profibrotic EVs secretion in macrophages, with Alix playing a more prominent role in this process.

### 2.6 Inhibition of Alix Can Alleviate LPS‐Induced EVs Secretion and SAPF Process

**Figure 6:**
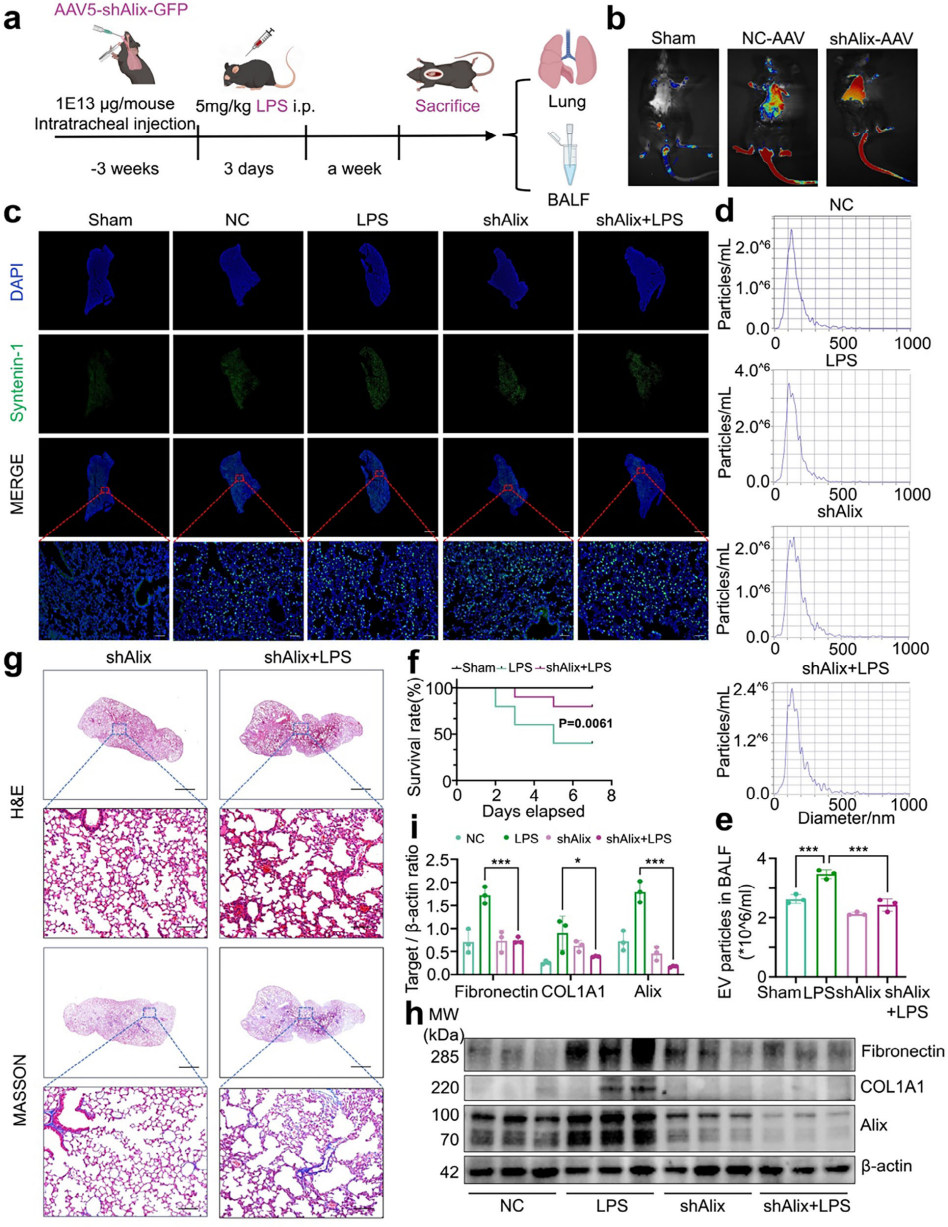
In vivo inhibition of Alix reduces EVs secretion and alleviates SAPF. a) Schematic illustrating Alix knockdown in LPS‐induced pulmonary fibrosis. Each mouse received an intratracheal injection of 1E13 µg adeno‐associated virus 5 (AAV5) expressing shAlix, followed by intraperitoneal LPS injection 3 weeks later. Mice were sacrificed 1 week after the LPS injection. b) The fluorescence of shRNA‐GFP was detected by an in vivo optical molecular imaging system (IMAGING 200, Raycision Medical Technology CO., Ltd., China). c) The transfection efficiency of vector‐AAV or shAlix‐AAV delivered via intratracheal injection was confirmed by detecting GFP signals using a fluorescence microscope. Scale bar=1500/200 µm (magnified view). d,e) NTA and corresponding quantitative analysis were performed to evaluate the size distribution and quantity of EVs in BALF from sham and LPS‐treated mice w/wo Alix knockdown (Mean ± s.d., ^***^ *P*< 0.001, One‐Way ANOVA, n = 3). f) Kaplan–Meier analysis of survival rate in LPS‐induced mice w/wo Alix knockdown. n = 10. g) H&E and Masson staining of LPS‐treated lung tissues, w/wo Alix knockdown. The images are representative of more than three mice per group. Scale bar=1500/100 µm (magnified view). h,i) Western blot and the corresponding quantitative analysis to assess Fibronectin and COL1A1 levels. (Mean ± s.d., ^*^ *P*< 0.05, ^***^ *P*< 0.001, Two‐Way ANOVA, n = 3).

To determine the role of Alix in the progression of SAPF, we generated adeno‐associated viruses 5 (AAV5) vectors expressing both Alix short hairpin RNA (shRNA) and GFP. The virus were delivered intratracheally into the lungs of the mice. Three weeks later, LPS was administered intraperitoneally for three consecutive days, and mice were sacrificed one week after the final injection (**Figure** **6**a). Successful lung targeting by shNC‐GFP and shAlix‐GFP was confirmed by GFP fluorescence, which was predominantly detected in the lungs (Figure 6b,c). We collected mouse BALF, performed ultracentrifugation, and conducted NTA to measure the number of EVs. The results demonstrated that Alix knockdown reduced EVs secretion in the BALF compared to the LPS group, highlighting Alix as an important driver of EV biogenesis in vivo (Figure 6d,e).

We next evaluated whether Alix silencing could mitigate pulmonary fibrosis and reduce LPS‐associated mortality. In line with our hypothesis, the mortality rate in LPS‐treated mice reached 60% by day 5, whereas Alix knockdown reduced this rate to 20% (Figure 6f). Additionally, Alix knockdown substantially mitigated LPS‐induced pulmonary fibrosis, as indicated by reduced collagen deposition and decreased alveolitis (Figure 6g). This effect was accompanied by diminished activation of lung fibroblasts, characterized by reduced expression of fibronectin and COL1A1(Figure 6h,i; Figure S5h, Supporting Information). Taken together, these findings indicate that Alix, a key regulator of EVs biogenesis, plays a pivotal role in the progression of SAPF. Targeting Alix effectively alleviates LPS‐induced EVs secretion, fibrosis, and associated mortality.

## 3 Discussion

In this study, we investigated the mechanism underlying the progression of SAPF. While the roles of glycometabolic reprogramming, profibrotic extracellular vesicles (EVs), and integrin activation in pulmonary fibrosis are well established, the connections among these factors remain unclear.^[^ ^32^, ^33^, ^34^, ^35^ ^]^ We propose a novel mechanism in which succinic acid, the product of glycometabolic reprogramming, induces macrophage endocytosis, leading to the release of profibrotic EVs. These EVs mediate the transfer of ITGβ1 between macrophages and fibroblasts, driving fibroblast activation and the progression of SAPF. Our findings suggest that profibrotic EVs, particularly ITGβ1 located on EVs membranes, represent a promising therapeutic target for SAPF.

Pulmonary fibrosis is characterized by the activation of pulmonary fibroblasts and ECM deposited in the pulmonary interstitial, leading to structural and functional disruption.^[^ ^36^ ^]^ SAPF is a critical clinical illness that poses a severe threat to life.^[^ ^37^ ^]^ Current research highlights that fibroblasts and myofibroblasts are key effector cells in ECM synthesis and pulmonary fibrosis progression.^[^ ^9^ ^]^ Metabolic reprogramming is increasingly recognized as a hallmark of fibrosis across many organs and cell types. Over the past decade, studies have revealed that shifts between glycolysis and fatty acid oxidation synergistically regulate ECM deposition.^[^ ^32^ ^]^ Interestingly, the bleomycin (BLM)‐induced pulmonary fibrosis mouse model also demonstrated elevated glycolysis and a disrupted tricarboxylic acid (TCA) cycle.^[^ ^38^, ^39^ ^]^ Through metabonomics analysis of TCA cycle intermediates in SAPF mouse lungs, we observed elevated levels of succinic acid, cis‐aconitic acid and l‐lactic acid. We have previously found that LPS can induce lactate accumulation and epithelial‐mecenchymal transition (EMT), the key regulatory mechanism of pulmonary fibrosis.^[^ ^40^ ^]^ Among these, succinic acid is particularly noteworthy as it has been identified as a critical mediator in local stress, ischemia, and hypoxia.^[^ ^41^, ^42^ ^]^ This phenomenon was further validated using LC‐MS analysis. Therefore, we propose that succinic acid may contribute to the progression and increased mortality of SAPF.

Integrins are a large family of transmembrane glycoproteins that act as cell adhesion receptors, consisting of one α subunit and β subunit joined by non‐covalent interactions.^[^ ^43^ ^]^ Our previous work revealed that integrin β3 in the lung fibroblasts is upregulated in response to mechanical ventilation (MV) and LPS‐induced pulmonary fibrosis and is further elevated by MV and LPS combination.^[^ ^44^ ^]^ The intercellular transfer of profibrotic EVs represents a critical component of disease pathogenesis. We have also demonstrated that the release of profibrotic EVs, mediated by ASK1‐ER stress, plays a key role in the interaction between alveolar epithelial cells and pulmonary fibroblasts during MV‐induced pulmonary fibrosis.^[^ ^45^ ^]^ Consistently, our present study shows that ITGβ1 transfer, mediated by profibrotic EVs, represents a novel mechanism underlying fibroblast activation and the progression of SAPF.

Endocytosis, the progress by which cells internalize plasma membrane components along with extracellular materials, is essential for both physiological and pathological functions. Clathrin‐mediated endocytosis involves the uptake of extracellular substances through clathrin‐coated vesicles and plays a pivotal role in exosome synthesis.^[^ ^46^, ^47^ ^]^ Integrins regulate both biosynthetic pathway outward transport and secretory vesicle exocytosis during this process.^[^ ^48^ ^]^ Our findings emphasize the critical role of succinic acid in inducing macrophages endocytosis, thereby promoting ITGβ1 transfer via profibrotic EVs release and driving SAPF progression.

Exosomes are formed as ILVs within MVBs. Syntenin‐1 is considered to interact with several binding partners that are involved in the synthesis and secretion of exosomes.^[^ ^49^ ^]^ Syntenin‐1 interact with Alix, a component of the ESCRT machinery, to facilitate membrane budding and scission.^[^ ^50^ ^]^ Studies have revealed that succinic acid can enhance the secretion of intestinal exosomes, further supporting the notion that succinic acid may play a role in the regulation of clathrin and Alix/Syntenin‐1 in the biogenesis of MVBs.^[^ ^48^ ^]^ Consistent with these studies, our work highlights the roles of Syntenin‐1 and Alix in the formation of macrophage‐derived profibrotic EVs. Furthermore, using adeno‐associated viral (AAV) vectors to knock down Alix expression via shRNA can effectively mitigate SAPF progression.

While our findings shed light on the ITGβ1 transfer mediated by the release of profibrotic EVs during SAPF, several aspects remain to be explored. For instance, integrins are composed of both α subunit and β subunit but our focus was limited to ITGβ1 due to the complexity of numerous α and β subunits dimer combinations. Thus, targeting both α and β subunits simultaneously through genetic inactivation is particularly challenging.^[^ ^51^ ^]^ Moreover, while we demonstrated that macrophages‐derived profibrotic EVs promote ITGβ1 transfer to activate fibroblasts, we did not investigate the effect of integrins activation originating from fibroblasts themselves. The detailed mechanism underlying these processes warrant further exploration.

## 4 Conclusion

In summary, our study demonstrates that succinic acid induces macrophages endocytosis, leading to the release of profibrotic EVs that mediate ITGβ1 transfer and subsequently activate fibroblasts during SAPF. Targeting ITGβ1 or inhibiting the release of profibrotic EVs effectively mitigates the progression of pulmonary fibrosis, offering promising molecular targets and therapeutic strategies for SAPF.

## 5 Experimental Section

### Ethics Statement and Animals

Male C57BL/6 mice (6–8 weeks old; 25–30 g) were purchased from Shanghai SLAC Laboratory Animal Co., Ltd., China. The study was approved by the Ethics Committee of Ren Ji Hospital, Shanghai Jiao Tong University School of Medicine (Approval No. RJ 2022‐0610). The mice were randomly and evenly divided into groups according to the experimental requirements and housed under specific‐pathogen‐free conditions. They were kept in a controlled environment with a 12‐h light/dark cycle and a temperature of 22–24 °C. The SAPF model was established by intraperitoneally injecting LPS (5 mg/kg, Escherichia coli O127, Sigma, USA) for three consecutive days, and mice were sacrificed after an additional week.^[^ ^40^ ^]^ GLPG0187 (100 mg/kg, MedChemExpress, HY‐100506) was intraperitoneally administered daily for three days, one hour prior to LPS injection. Mice were sacrificed after one week, and the lungs and bronchoalveolar lavage fluid (BALF) samples were subsequently collected for further investigations.

### Cell Culture

Raw264.7 cells, HEK293T cells, and 3T3‐L1 cells were cultured in Dulbecco's modified Eagle's medium (DMEM; BasalMedia, L110KJ) supplemented with 10% fetal bovine serum (FBS, Gibco, USA), 100 IU/mL penicillin, and 100 IU/mL streptomycin. The complete medium was ultracentrifuged at 100000 × g for 70 min and filtered through a 0.22 µm membrane to remove EVs contamination from the FBS. These cell lines were cultured in a 5% carbon dioxide incubator at 37 °C and passaged after trypsinization.

### Inhibition by ShRNA‐AAV Injection

Adeno‐associated virus serotype 5 (AAV5) was obtained from Genomeditech (Shanghai, China) and used to express shAlix or vector. Under anesthesia, mice underwent tracheotomy and were injected with 50 µL of PBS containing 1E13 µg of virus per mouse. Three weeks later, they subsequently received an intraperitoneal injection of LPS (Figure 6a).

### Lung Histopathology and Immunofluorescence

Lung tissues were excised, fixed in 4% paraformaldehyde (PFA) for 48 h, embedded in paraffin, and sectioned into 5‐µm‐thick slices. Lung sections were stained with H&E staining to visualize inflammatory cells infiltration in the alveoli and pulmonary interstitium, and Masson's trichrome to identify fibrotic foci, following the manufacturer's instructions.

Tissue immunofluorescence dual staining was employed to evaluate the expression levels and localization of COL1A1, ITGβ1, Clathrin, F4/80, Alix, and Syntenin‐1. Briefly, paraffin sections were rinsed three times with PBS for 5 min, blocked with 10% BSA for 1 h, and incubated with primary antibodies against COL1A1 (72026S, CST, USA), ITGβ1 (34971S, CST, USA), Clathrin (4796T, CST, USA), F4/80 (sc‐52664, Santa cruz, USA), Alix (12422‐1‐AP, Proteintech, USA), Syntenin‐1 (22399‐1‐AP, Proteintech, USA) at a dilution of 1:100 overnight at 4 °C. This was followed by incubation with Alexa Fluor 594‐conjugated anti‐mouse IgG and Alexa Fluor 488/594‐conjugated anti‐rabbit IgG (A‐11005, A‐11008/A‐11012, Thermo Fisher, USA) and counterstaining with DAPI (Sigma Chemicals) at the end. Photographs of the sections were captured using a laser scanning confocal microscope (Olmpus, FV3000, Japan).

### Extraction of Mouse BALF and EVs

BALF was collected from anesthetized mice by performing a tracheotomy to expose the trachea. A tracheal catheter was then inserted, and 0.7 mL of PBS was injected twice to lavage the lungs. BALF samples were first centrifuged at 300 × g for 10 min to remove cells and cellular debris, followed by centrifugation of the supernatants at 2000 × g for 10 min to eliminate dead cells. Subsequently, the supernatants were centrifuged at 10 000 × g for 30 min to further remove large vesicles and cellular debris. Finally, the resulting supernatants were centrifuged at 100 000 × g for 70 min, twice, at 4 °C to pellet EVs. The EV pellets were resuspended in 200 µL of 0.1 µm filtered 1 × PBS for further analysis.^[^ ^52^ ^]^

### Flow Cytometry Analysis

The enzymatic digestion of the lung tissues was performed using a pulmonary dissociation kit (130‐095‐927, Miltenyi Biotec, Germany). For mechanical dissociation, a specialized pulmonary tissue dissociater was utilized, and the resulting cell suspension was filtered through a 70 µm filter and subsequently centrifuged. The supernatant was completely removed, and the cells were then resuspended in DPBS for further experiments.

The single cell suspensions were preincubated with Fc Block purified anti‐mouse CD16/CD32 mAb (553141, BD, USA) and then was incubated with fluorochrome‐conjugated antibodies targeting CD45, CD11b, and F4/80. After washing with DPBS, the cells were analyzed using FACS Verse flow cytometry (BD). The data were processed with FlowJo software (version 10) for analysis.

### Isolating and Labeling EVs from Macrophages

Raw264.7 cells were treated with 5 mM succinic acid (S3791, Selleck, USA) for 48 h to obtain conditioned culture medium containing profibrotic EVs. Subsequently, EVs were purified from the serum‐free DMEM medium following the manufacturer's instructions. As described previously, the medium was subjected to multiple rounds of gradient ultracentrifugation, and the supernatants were filtered through a 0.22 µm membrane to further purify EVs, which were then resuspended in 200 µL of 0.1 µm filtered PBS. The 20 µL of the re‐suspensioned EVs was quantified using a bicinchoninic acid (BCA) protein assay kit (23225, Thermo Fisher, USA). Then the corresponding volume of EVs was calculated based on the protein concentrations of the EVs (e.g., 40 µg) to further act on fibroblasts.

To investigate the EVs uptake process, EVs were labeled with PKH67 green fluorescent (PKH67GL, Sigma–Aldrich, USA) or PKH26 red fluorescent (PKH26GL, Sigma–Aldrich, USA), which incorporates into the EV membranes. First, 100 µL of EVs were mixed with 500 µL of Dilution C, while 4 µL of PKH67 or PKH26 was simultaneously mixed with 500 µL of Dilution C. The EVs solution was gently combined with the PKH solution, mixed for 30 seconds, and incubated at room temperature for 5 min. The process was terminated by adding 1 mL of 0.5% BSA, followed by ultracentrifugation at 110000 × g for 2 h to pellet fluorescently labeled EVs.

### EVs Uptake by Fibroblasts

To examine the uptake of profibrotic EVs by fibroblasts, the cell line 3T3‐L1 was seeded on coverslips in 24‐wells or 12‐well plates for subsequent immunofluorescence (IF) and western blot experiments. It should be noted that the specific volume of PKH‐labeled EVs to be added was determined based on the results of BCA protein quantification and the specific experimental requirements. For western blot experiments, cells were stimulated with EVs for 24 h before protein lysates were isolated for subsequent analysis. However, for IF experiments, cells were either treated for short durations of 30 and 180 min, or for longer durations of 24 h before fixation.

### Nanoparticle Tracking Analysis (NTA) of EVs

The resuspended EVs were analyzed for the purity, particle size distribution, and concentration using the nanoparticle tracking analysis (NTA) technology (Particle Metrix, Zetaview‐PMX120‐Z, Germany). The instrument was calibrated with polystyrene microspheres (100 nm), and the sample chamber was cleaned with 1 × PBS. The EVs pellet was diluted with 1 × PBS to improve the accuracy of the analysis. Finally, the data were processed and analyzed using the ZetaView software (version 8.05.14 SP7).

### Transmission Electron Microscopy (TEM)

To observe the specific morphology of EVs, the sample was loaded on a formvar film (BZ11262a, Zhongjing, China) and incubated for 5 min. The excess liquid was removed using filter paper. The grid was washed with distilled water and subjected to negative staining with 2% uranyl acetate on a formvar film stabilized with a carbon support layer (02624‐AB, Ted Pella Inc, USA), and the remaining solution was removed again. The EVs were observed using a TEM (HT7800, HITACHI, Japan).

To examine the quantity and structure of MVBs, Raw264.7 cells treated with succinic acid or control cells were first centrifuged, washed twice with 1 mL of PBS, and then fixed in 2.5% glutaraldehyde at 4 °C for 24 h. Following fixation, the sample was embedded in 1% agarose, fixed with 1% OsO_4_ in 0.1 M PBS for 2 h at room temperature, and rinsed in PBS three times for 15 min each.

The samples were then dehydrated through a graded ethanol series for 10 min each and subsequently embedded in resin. Ultrathin sections were prepared, stained with 2% uranyl acetate saturated alcohol solution for 10 min, and visualized using a TEM as previously described.

### LC‐MS Succinic Acid Analysis

The succinic acid levels in mouse lung tissues and BALF were determined using a specific liquid chromatography‐mass spectrometry (LC‐MS) technique, namely Ultra‐Performance Liquid Chromatography‐Triple Quadrupole Mass Spectrometer (TQ‐XS) (Waters, Milford, MA, USA).

### Plasmids

A Halo‐tagged mouse ITGβ1 construct containing nine amino acid flanking linkers was cloned into pSLenti‐CMV‐Itgb1 (p.1‐101aa)‐EFGGSGGSG‐Halo‐ GGSGGSGLE‐Itgb1 (p.102‐798aa)‐PGK‐Puro‐WPRE. Additionally, the plasmid pSLenti‐CMV‐Itgb1‐linker‐mCherry‐3xFLAG‐PGK‐Puro‐WPRE was purchased from Obio Technology (Shanghai) Corp., Ltd.

### Lentivirus Generation and Cell Infection

HEK293T cells were plated in 6 cm dishes and transfected with 1 µg of shRNA targeting *ITGβ1* (with the targeting sequence GCACGATGTGATGATTTAGAA), *Alix* (with the targeting sequence CGAGGTTGTAAGTGTCTTAAA), or Syntenin‐1 (with the targeting sequence CGAACACATTATTAAACGGAT). Transfections were performed using the pSLenti‐U6‐shRNA‐CMV‐EGFP‐F2A‐Puro‐WPRE lentiviral vector, together with 1 µg of the packaging plasmid (psPAX2) and 1 µg of the envelope plasmid (pMD2.G), using the Polyjet transfection reagent (SL100688, SignaGen, USA). After 16 h, the medium was replaced with fresh growth medium, and the cells were incubated for an additional 24 h. Following incubation, the viral supernatant was harvested and filtered through a 0.45 µm filter. To infect Raw264.7 cells, 1 mL of the filtered viral supernatant was mixed with 1 mL of complete growth medium and 2 µL of polybrene. The mixture was then applied to the cells for 24 h. Infected cells were selected with 5 µg/mL puromycin for 36–48 h to establish stable cell lines.

### Western Blot

To detect protein expression levels in mouse lung tissues, 50 mg of fresh lung tissues were weighed, and 500 µL of RIPA lysis buffer (P0013D, Beyotime, China) was added. The tissue was thoroughly homogenized using a high‐speed tissue homogenizer to obtain lung tissue homogenates.

For protein expression analysis in EVs and cells, proteins were extracted and lysed using ice‐cold RIPA buffer. The lysates’ protein was measured using a BCA protein assay kit. Subsequently, 30 µg of protein was loaded onto 12% SDS‐PAGE gels for separation, transferred onto a 0.45 µm polyvinylidene fluoride (PVDF) membrane (IPVH00010, Millipore, USA), and blocked with a protein‐free rapid blocking buffer (PS108, Epizyme, China). The membranes were then incubated overnight at 4 °C overnight with primary antibodies, including anti‐COL1A1 (72026S, CST, USA), anti‐ITGβ1 (34971S, CST, USA), anti‐Fibronectin (26836S, CST, USA), anti‐Alix (12422‐1‐AP, Proteintech, USA), anti‐Syntenin‐1 (22399‐1‐AP, Proteintech, USA), anti‐CD63 (A19023, Abclonal, China), anti‐CD9 (A19027, Abclonal, China), anti‐Na, K‐ATPase (3010S, CTS, USA), anti‐TSG101 (A1692, Abclonal, China), and anti‐β‐actin (AC026, Abclonal, China).

### Live Cell Imaging

To show that ITGβ1 is transferred to fibroblasts via EVs released by macrophages, a Halo system was constructed to trace the uptake of EVs. Briefly, RAW264.7 cells were transduced with lentivirus to generate a stable cell line overexpressing HaloTag‐tagged ITGβ1 and subsequently collected the released EVs. After quantifying the EVs protein content, it labeled them with PKH26 red fluorescence and used them to stimulate 3T3‐L1 cells for 24 h at the required doses. The Halo ligand was then added, Oregon Green permeability dye (G2801, Promega, USA) diluted in pre‐warmed culture medium, to 24‐well plate and incubated them in a cell culture incubator for 15 min. After washing the wells three times with pre‐warmed complete medium (without phenol red), the plate was incubated for an additional 30 min in the incubator. Following this, the cells were rinsed with PBS, fixed, permeabilized, and labeled with phalloidin (40762ES, yeasen, China) to observe the images under a laser scanning confocal microscope.

### Cell Immunofluorescence

3T3‐L1 cells and primary fibroblasts were plated on glass coverslips in a 24‐well plate. After attachment, the cells were fixed with 4% paraformaldehyde for 15 min and permeabilized with Triton X‐100 (P0096, Beyotime, China) for 8 min at room temperature. Following permeabilization, the cells were blocked with staining blocking buffer (P0102, Beyotime, China) for 30 min and then incubated with primary antibodies, as previously described, at room temperature for 1 h. The cells were washed twice with PBST and subsequently incubated for 1 h with Alexa Fluor 555‐conjugated anti‐rabbit IgG, Alexa Fluor 488‐conjugated anti‐mouse IgG (A‐21428, A‐11001, Thermo Fisher, USA), and iFluor 647 phalloidin.

### Primary Fibroblast Isolation

Mice were anesthetized and the surgical area was sterilized with alcohol. An incision was made in the thoracic cavity to remove the lung lobes. The excised lung tissues were rinsed with sterile PBS, cut into fragments, and digested with 0.1% collagenase (10837091001, Roche, Switzerland) for 1 h. The digested tissues were then washed with sterile PBS and centrifuged at 1500 rpm for 10 min. The resulting tissue suspension was resuspended in DMEM medium supplemented with 15% FBS and 5% penicillin‐streptomycin solution. After 4–5 days of culture, adherent fibroblasts were collected for further passaging or downstream assays.

### Single‐Cell Analysis

scRNA‐Seq was conducted on lung cells from the sham (n = 3) and LPS (n = 3) groups. The tissue samples were prepared and preprocessed following the protocol outlined in the previous work.^[^ ^53^ ^]^ Genes expressed in at least three cells were included for further analysis. Barcoded sequencing libraries were prepared using 10 × Genomics (Biomarker Technologies, China). Following initial quality control, cells were retained based on stringent criteria (*nFeature_RNA*: 200‐4500; mitochondrial gene proportion, *percent.mt*: < 5%). Data normalization was performed, and 2000 highly variable genes were identified using the variance stabilization (“vst”) method, followed by z‐score scaling. Principal component analysis (PCA) was applied for dimensionality reduction, and the top 15 principal components were selected for downstream clustering. Uniform Manifold Approximation and Projection (UMAP) was employed to visualize the resulting cell clusters. Cell type annotation was conducted by combining SingleR computational predictions with manual validation using canonical marker genes for mouse lung tissues, including MS4A1 and CD79A (B cells), CD3E and CD8A (T cells), as well as CD14 and LYZ (monocytes). Cell–Cell communication networks were reconstructed computationally using CellChat (v2.1.0), based on the mouse ligand‐receptor interaction database (CellChatDB.mouse). Normalized gene expression matrices were analyzed to identify overexpressed ligand‐receptor pairs using the trimmed mean threshold (set at 10%) with the *truncatedMean* method. Differential signaling between groups was assessed using merged CellChat objects. To perform Gene Ontology (GO) and Kyoto Encyclopedia of Genes and Genomes (KEGG) pathway enrichment analysis, differentially expressed genes (DEGs) were identified for specific clusters or cell types based on statistical criteria (p‐value < 0.05, log2FC> 1.5). All data analysis and visualization were carried out in R (v4.4.2).

### Quantitative Proteomics Identified Proteins Enriched in EVs

EVs obtained by ultracentrifugation were treated with RIPA buffer and PMSF to a final concentration of 1 mM, followed by ultrasonic disruption on ice for 2 min at 80 W. The solution was centrifuged at 12000 rpm for 10 min at 4 °C, and the supernatant was collected. This process was repeated twice to obtain the total protein solution. Protein concentration was measured using a BCA assay kitmM for subsequent analysis. The samples were subjected to proteolysis and peptide desalting, separated using an Ultra‐High‐Performance Liquid Chromatography (UHPLC) system, and analyzed on a timsTOF Pro mass spectrometer (Bruker, Germany). Data‐Independent Acquisition (DIA) raw data were processed using Spectronaut Pulsar 18.4 software (Biognosys, Switzerland). Differentially expressed genes (DEGs) were identified based on statistical thresholds (p‐value < 0.05, fold change> 1.2 or < 0.83), and data analysis was performed on the OECloud platform (https://cloud.oebiotech.cn/).

### Statistical Analysis

Data analysis was conducted using GraphPad Prism 10 (Version 10.1.1). An unpaired two‐tailed *t*‐test was used for comparisons between two groups, while one‐way or two‐way ANOVA was applied for multiple group comparisons, as appropriate. Kaplan–Meier survival rate analysis was employed to evaluate survival data. Data were presented as mean ± s.d., with significant levels denoted as “ns” indicating not significant, ^*^ *P*< 0.05, ^**^ *P*< 0.01, ^***^ *P*< 0.001.

## Conflict of Interest

The authors declare no conflict of interest.

## Author Contributions

W.Y. and R.T. contributed equally to this work. W. Y., Q. X., and Z. H. Conceived and designed the experiments; W.Y., R. T., Y. P., and Y. Z. Performed the experiments; S. M. and X. H. assisted in experiments; W. Y. and J. Z. analyzed and interpreted the data; J. Z., W. Y., and R. T. performed scRNA‐seq data analysis; S. X. and Y. G. administrative support; W. Y., Q. X., and Z. H. wrote the manuscript.

## Supporting information



Supporting Information

## Data Availability

The data that support the findings of this study are available from the corresponding author upon reasonable request.
