# Supplementary material for: Succinic Acid‐Induced Macrophage Endocytosis Promotes Extracellular Vesicle‐Based Integrin Beta1 Transfer Accelerating Fibroblast Activation and Sepsis‐Associated Pulmonary Fibrosis
Source: Adv Sci (Weinh). 2025 Sep 2;12(43):e07411. doi: 10.1002/advs.202507411 (PMC12631846; doi:10.1002/advs.202507411)
Supplement: Supplementary file 1 — Supporting Information [file ADVS-12-e07411-s001.docx]

**
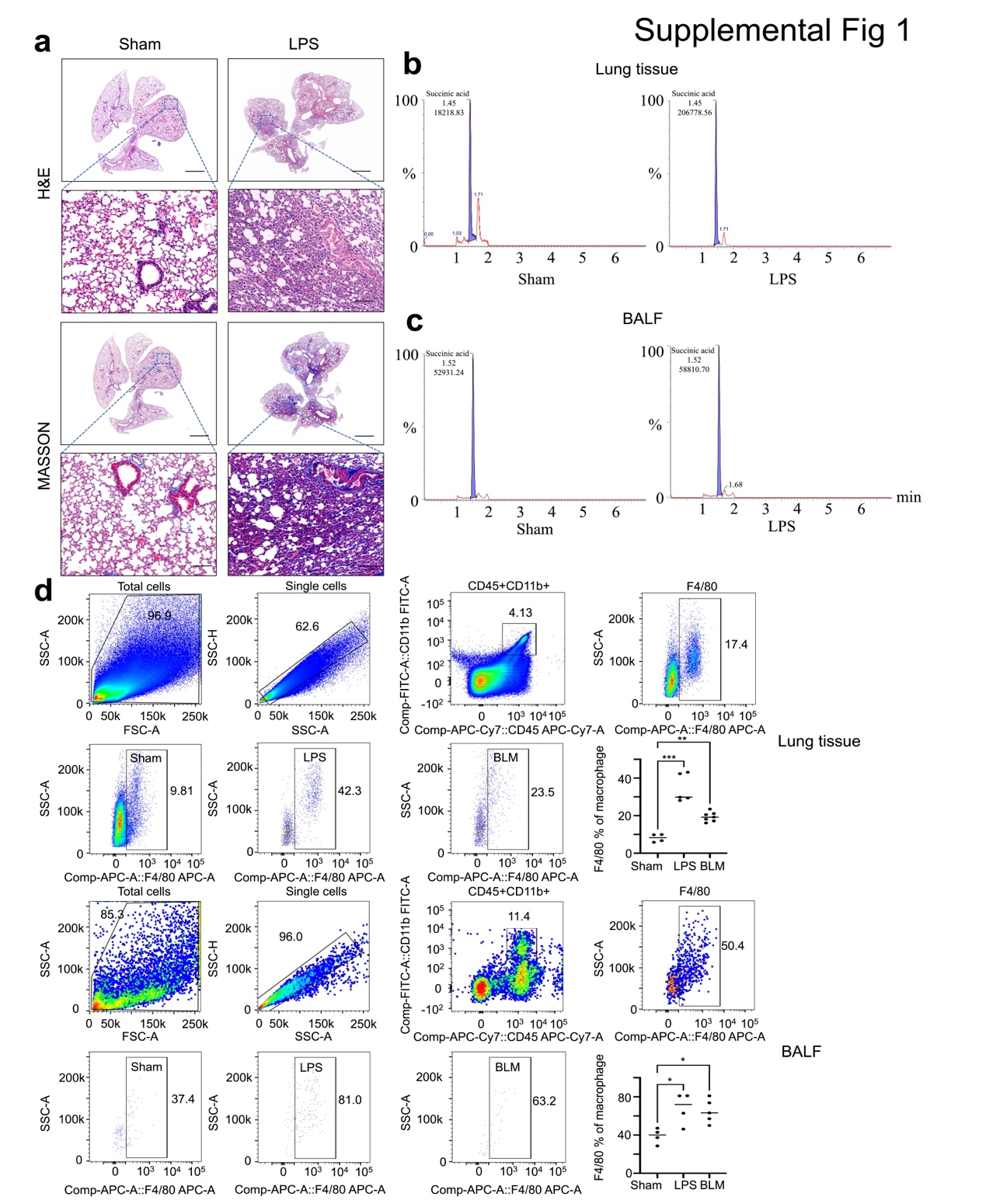
Supplemental Figure Legends**

**Figure S1. SAPF was induced by LPS and macrophages play a crucial role in the secretion of EVs and the regulation of succinic acid metabolism. (a)** H&E and Masson staining of sham and LPS groups. The images are representative of more than three mice per group. Scale bar=1500 µm/100 µm (magnified view). **(b-c)** LC-MS peak profile schematic for detecting succinic acid concentration in lung tissues (n=3) and BALF (n=5) from sham and LPS groups. **(d)** Macrophages isolated from mouse lung and BALF samples were stained with CD45, CD11b and F4/80 and frequencies of macrophages were measured by flow cytometry. Example for basic gating strategy and bar plots representing the percentage of F4/80^+^ cells in murine lung and BALF. (Mean ± s.d., *P<0.05, **P<0.01, ***P<0.001, One-Way ANOVA).

**
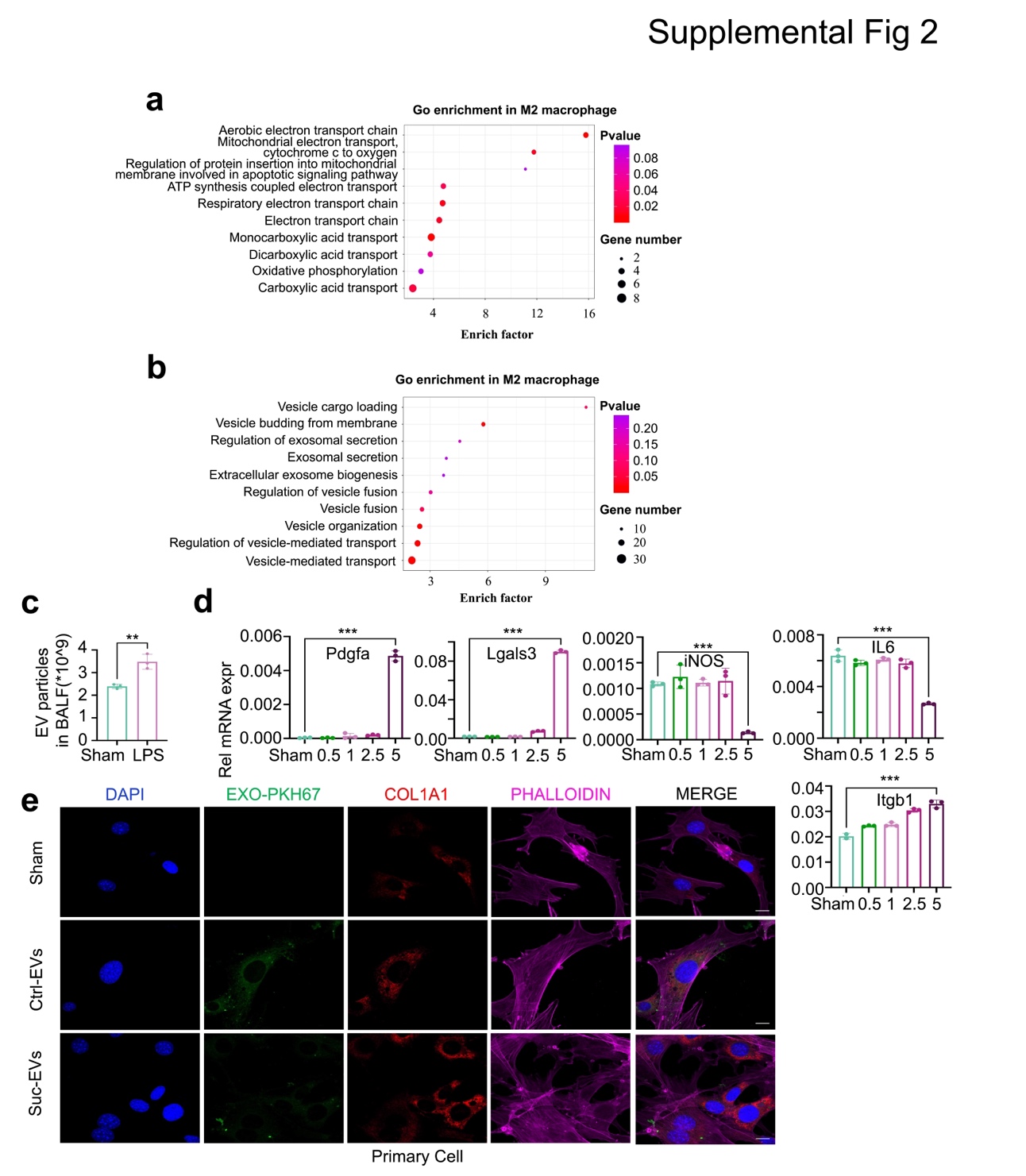
Figure S2. Profibrotic EVs secretion in macrophages stimulated by Succinic acid. (a-b)** GO analysis of upregulated DEGs derived from M2 macrophages in the lung tissues of mice stimulated with LPS. **(c)** Enzyme-linked immunosorbent assay (ELISA) was used to detect the number of EVs particles in mouse BALF. **(d)** RT-PCR analysis to determine the optimal succinic acid concentration for stimulating macrophages to produce profibrotic EVs, with all stimulation durations set to 48h. (Mean ± s.d., ***P<0.001, One-Way ANOVA, n=3). **(e)** Immunofluorescence analysis of COL1A1 in primary fibroblasts stimulated with 40 µg EVs. **
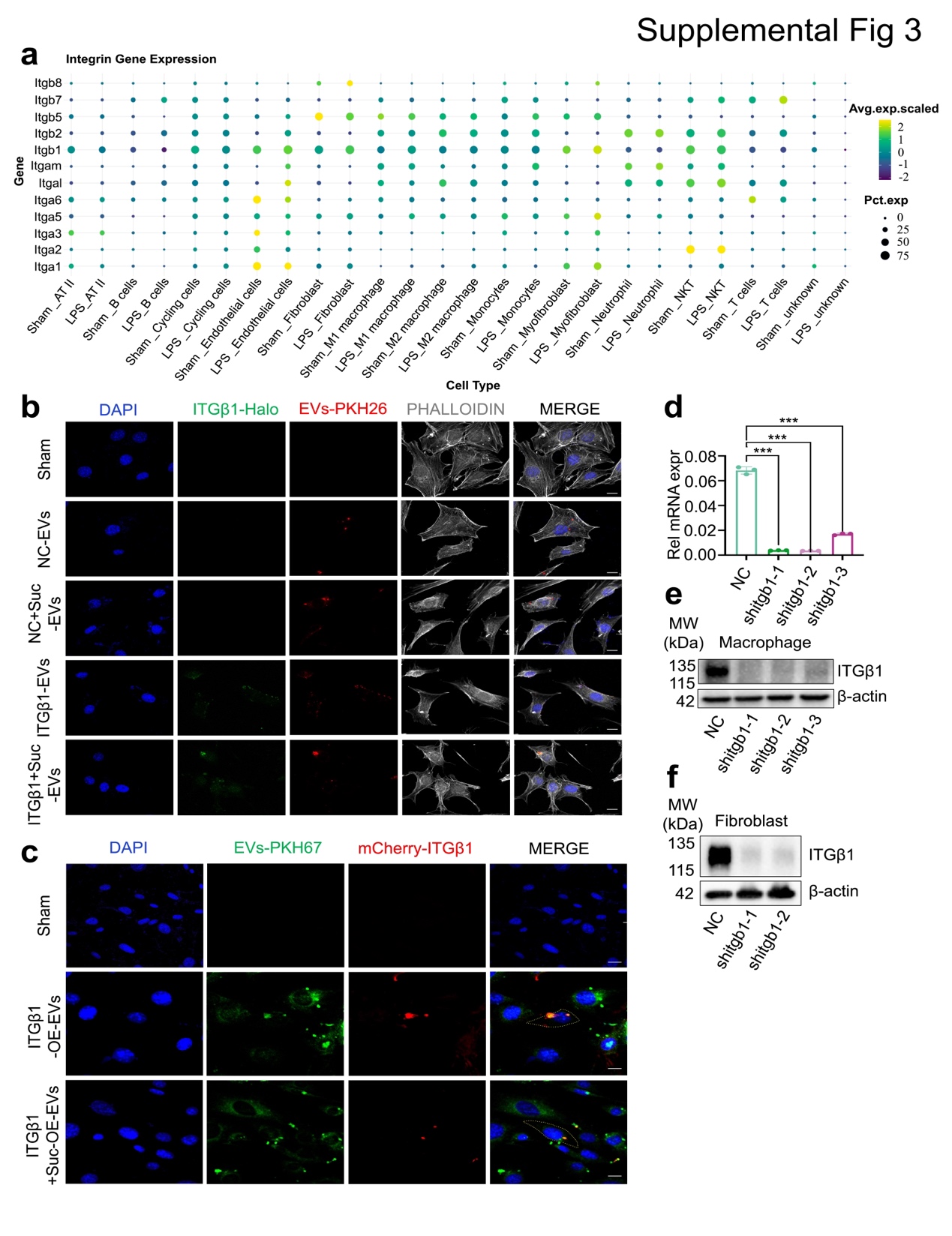
**Scale bar=10 µm.

**Figure S3. EVs-mediated ITGβ1 transfer in macrophages is a key factor for fibroblast activation. (a)** A dotplot illustrating the expression levels of integrin family members across various cell subpopulations within mouse single cell dataset. **(b)** Overlay image of fibroblasts reconstituted with ecto-Halo ITGβ1, after sequential labeling with HaloTag Oregon Green Ligand and imaged by laser scanning confocal microscope. PKH26-labeled EVs appeared as red punctate structures, Halo-labeled ITGβ1 appeared as green punctate structures and phalloidin (white)-labeled F-actin represents the cell outline. Scale bar=10 µm. **(c)** Overlay image of fibroblasts reconstituted with mCherry-ITGβ1, w/wo succinic acid treatment. PKH67-labeled EVs appeared as green punctate structures while mCherry-labeled ITGβ1 appeared as red punctate structures. Scale bar=10 µm. **(d-e)** RT-PCR and western blot analysis to validate the knockdown efficiency of Itgb1 in macrophages. (Mean ± s.d., ***P<0.001, One-Way ANOVA, n=3). **(f)** Western blot analysis to validate the knockdown efficiency of ITGβ1 in fibroblasts.


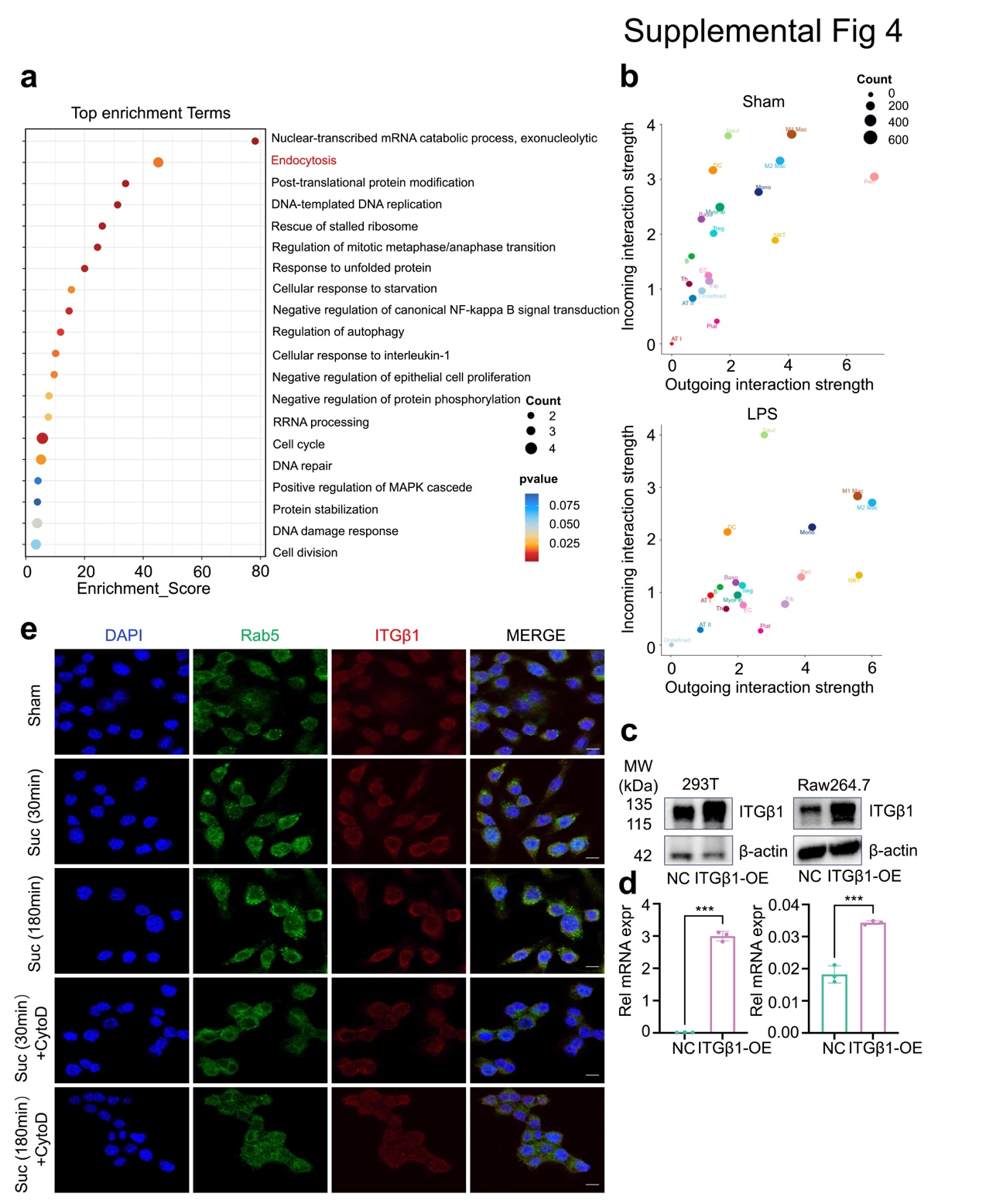
**Figure S4.** **ITGβ1 endocytosis promoted by succinic acid, is critical for MVBs formation.** **(a)** KEGG analysis of pathways enriched in EVs from macrophages treated w/wo succinic acid. **(b)** Signaling role analysis visualized the cell-cell communication network from single-cell sequencing data in lung tissues of sham and LPS groups. The x-axis and y-axis represent the total outgoing and incoming communication rates for each cell group, respectively. Dot color indicates cell groups and dot size is proportional to the inferred number of ligands and receptors for each group. **(c-d)** RT-PCR and western blot analysis to validate the overexpression efficiency of mCherry-Itgb1 in 293T cells and Raw264.7 cells. (Mean ± s.d., ***P<0.001, unpaired *t*-test, n=3). **(e)** Immunofluorescence analysis of MVBs marker Rab5 and ITGβ1 in macrophages stimulated with 5 mM succinic acid for 30 min or 180 min, w/wo cytoD pretreatment. Scale bar=10 µm.

**
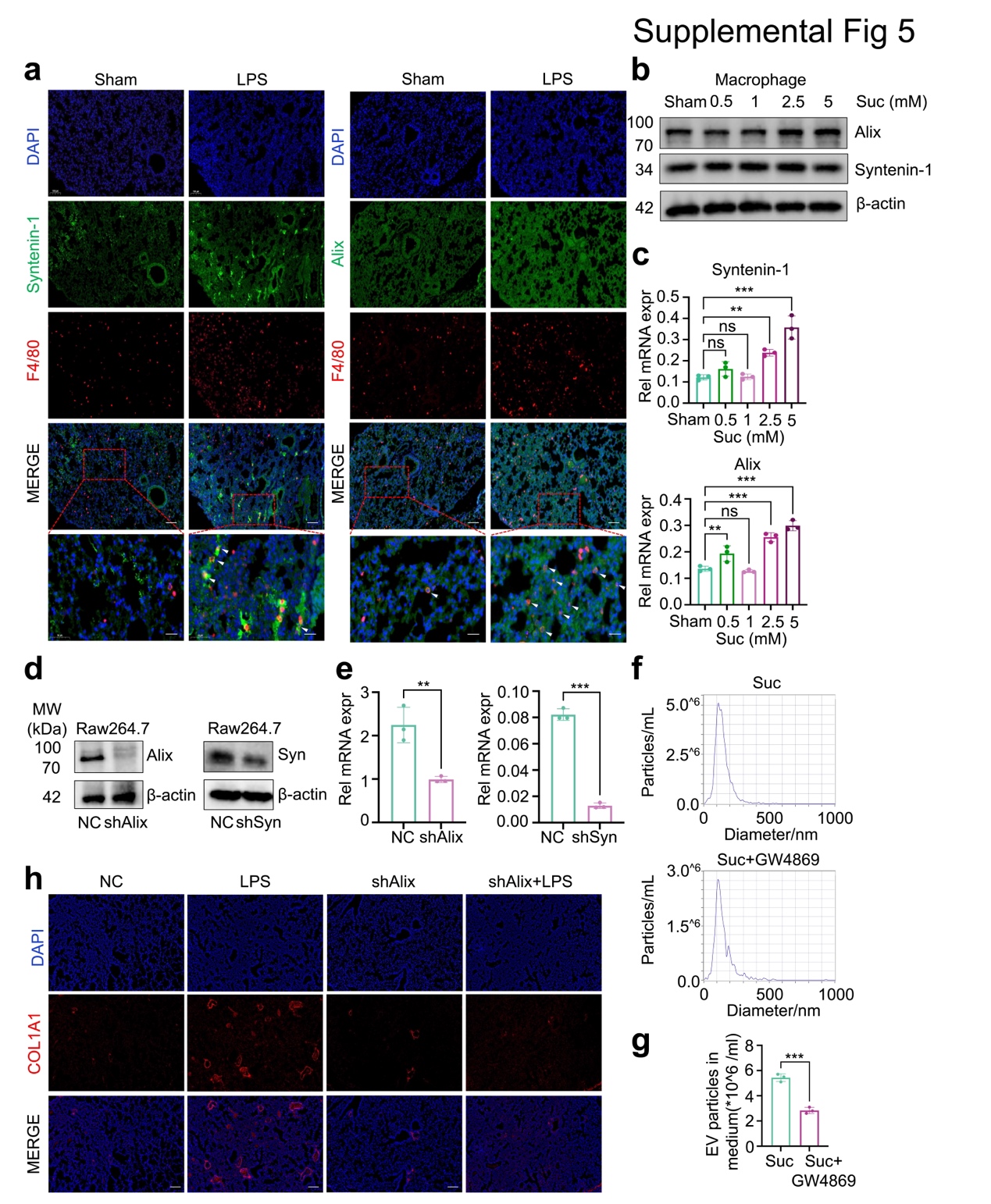
Figure S5. Alix, along with Syntenin-1, plays a key role in macrophages-fibroblasts interaction and fibroblasts activation. (a)** Immunofluorescence analysis of the relative expression and distribution of Syntenin-1, Alix and F4/80. White arrows indicate high expression of Syntenin-1 or Alix in macrophages. Scale bar=200 µm/30 µm (magnified view). **(b-c)** RT-PCR and western blot analysis to validate the expression levels of Syntenin-1 and Alix after stimulating macrophages with different concentrations of succinic acid (Mean ± s.d., **P<0.01, ***P<0.001, “ns” indicating not significant, One-Way ANOVA, n=3)**. (d-e)** RT-PCR and western blot analysis to validate the knockdown efficiency of Alix or Syntenin-1 in macrophages. (Mean ± s.d., **P<0.01, ***P<0.001, One-Way ANOVA, n=3). **(f-g)** NTA and corresponding quantitative analysis were performed to evaluate the quantity of EVs from Suc-EVs treated macrophages w/wo 10 µM GW4869 pretreated for 2h (Mean ± s.d., ***P<0.001, One-Way ANOVA, n=3). **(h)** Immunofluorescence analysis of lung tissues from the sham and LPS groups in mice, w/wo Alix knockdown, revealed the expression of COL1A1. Scale bar=200 µm.
